# Postmarital residence rules and transmission pathways in cultural hitchhiking

**DOI:** 10.1073/pnas.2322888121

**Published:** 2024-11-18

**Authors:** Simon Carrignon, Enrico R. Crema, Anne Kandler, Stephen Shennan

**Affiliations:** ^a^McDonald Institute for Archaeological Research, University of Cambridge, Cambridge CB2 3ER, United Kingdom; ^b^Department of Archaeology, University of Cambridge, Cambridge CB2 3DZ, United Kingdom; ^c^Department of Human Behavior, Ecology and Culture, Max Planck Institute for Evolutionary Anthropology, Leipzig 04103, Germany; ^d^Institute of Archaeology, University College London, London WC1H 0PY, United Kingdom

**Keywords:** cultural hitchhiking, sex-biased transmission, transmission pathways, postmarital residence rules, individual-based modeling

## Abstract

Cultural evolutionary processes can often lead to a statistical association between neutral and adaptive traits during episodes of population dispersal and the introduction of a beneficial technology in a geographic region. Here, we examine such cultural hitchhiking processes using an individual-based model that portrays the cultural interaction between a migrant and an incumbent population. Our model is loosely based on the interaction between farming and foraging populations during the initial stages of the adoption and diffusion of agricultural practices. The two populations are characterized by different variants for their neutral and adaptive cultural traits, with the latter set providing a reproductive advantage for the migrant communities over the incumbent ones. We explore how the neutral traits of the migrant population spread and how this process is conditioned by the following factors: 1) the possibility of transmission of the adaptive traits; 2) the extent of the increased reproductive advantage provided by the adaptive variants of the migrant population; 3) postmarital residence rules; and 4) how and when neutral traits are transmitted. Our results reveal a diverse range of outputs, highlighting the relevance of factors such as the nature of postmarital resocialization and the specific combination of postmarital residence rules and sex-biased transmission.

## Significance

Population dispersal and admixture can promote the spread of adaptive cultural traits, such as agriculture, in a geographic region. In some circumstances, this process can lead to the spread of selectively neutral traits that are transmitted together with adaptive ones, leading to what some refer to as cultural hitchhiking. Here, through an individual-based simulation, we demonstrate that factors such as postmarital residence rules, probability of postmarital social learning, sex-biased transmission, and transmission pathways can all impact the degree of hitchhiking and intergroup cultural diversity. Our results demonstrate how intergroup exogamy is a complex phenomenon that can theoretically lead to a substantially wide range of expected patterns.

In archaeology, migration and diffusion have been proposed as generic explanations for cultural change for well over a century, but the terms were usually used informally without any detailed specification of mechanisms or their effects. As such, while these processes have often been invoked to provide post hoc explanations of observed patterns in the archaeological record, formal descriptions of what we should expect to observe when specific mechanisms are in play were rarely developed. The foundations for going beyond the traditional view were only established in the 1980s. As part of their formulation of a quantitative approach to cultural transmission, Cavalli-Sforza and Feldman (1, pp.157–176) outlined a mathematical framework for examining how physical migration between two groups, or any sort of communication between them, could lead to changes in cultural trait frequencies in the groups through horizontal (between members of the same generation) or oblique (from members of the older to the younger generation) transmission, not least the introduction of innovations from one group to another.

Since the development of these theories and the emergence of the field of cultural evolution (1, 2), a large number of detailed ethnographic and ethnoarchaeological studies have revealed the complexity and specificity of cultural transmission in different domains (3–6). These differences become central when we investigate the social and biological processes that can lead to the spread of particular cultural variants.

In this paper, we focus on the processes involved in the spread of farming and the resulting interactions between migrant agricultural groups and incumbent foraging populations that have occurred in many parts of the world at different times. These key transitions have been the subject of a long-standing debate between those who have seen it as demographic dispersal resulting from higher reproductive success associated with the new subsistence economy possessed by the migrants and those who see it as a result of the adoption of farming as an innovation by local indigenous groups. These contrasting, but not mutually exclusive, views have very different implications for the transmission of other cultural traits associated with the different groups (7, 8). Their dynamics on the spread of farming can be generalized to a wider range of processes involving the interaction between two populations with different adaptive trait variants. Thus, given two communities in the same spatial region, a particular cultural variant held by one community might spread through this region purely because of differences in the growth rates between the two communities. Under such a scenario, the spread of a cultural variant does not involve genetic admixture or intergroup cultural transmission and is referred to as *demic diffusion*, or population dispersal. In contrast, the two communities may experience some form of intergroup cultural transmission via brief social interactions or through the movement of objects traded and exchanged, followed by emulative learning and reverse engineering (9). Under such a scenario, the spread of a cultural variant does not involve genetic admixture or population growth and is referred to as (pure) *cultural diffusion*. However, the spread of a cultural variant is rarely governed by pure demic or cultural diffusion and often entails some mixture of the two processes as well as genetic admixture. Indeed, cultural diffusion frequently entails repeated episodes of social interactions between individuals, often following episodes of migration where individuals permanently switch their community of residence. While the movement of individuals across cultural groups can be the result of a wide range of processes, in relatively small-scale societies, such as those associated with the transition to farming, the most common form is intergroup exogamy, i.e. the social practice of marrying outside one’s local group. These episodes of postmarital movement offer the most prominent pathway for cultural transmission across communities. However, intergroup exogamy can take different forms and have different (population-level) consequences. They commonly entail different postmarital residence rules and different opportunities for migrants to engage in cultural transmission. For example, female Luo potters in Kenya experience resocialization, learning their craft after marriage and change in residence from their mothers-in-law or senior cowives within a patrilocal society (10). This would lead to different patterns when contrasted to other forms of postmigration learning, for example, learning from both males and females in the community. The relevance of intermarriage and postmarital residence as a key vector of the spread of cultural variants has been, and continues to be, suggested in the archaeological literature since it is well documented in the ethnographic literature and provides a basis for the multiyear close-quarters interaction behind the transmission of many socially learned variants (4, 6, 11–13). However, theoretical expectations of what we should observe when specific assumptions and conditions are met remain a comparatively less researched area of investigation. Here, we aim to address this issue, focusing in particular on the interplay between postmarital residence rules and resocialization involving two populations with initially distinct adaptive variants. Our goal is to explicitly explore how these processes may lead to cultural hitchhiking and influence the spread of adaptive and nonadaptive cultural trait variants, therefore providing insights into the diffusion of agriculture.

## Cultural Hitchhiking and Diffusion of Beneficial Traits.

Human groups are often characterized by differences in adaptive traits that may result in different population growth rates. As noted above, different population growth rates alone can contribute to the success of a particular cultural variant in a region promoted by the higher growth rate of one particular group. However, in most cases, groups are not isolated from each other, and we often observe some degree of genetic admixture and cultural interaction. The spread of agriculture, which forms the focus of this paper, is perhaps the most emblematic example of this phenomenon, where migrant and expanding communities of farmers have interacted with incumbent groups of foragers. A large number of empirical and theoretical studies have examined the spread of agriculture, focusing, for instance, on the extent to which the diffusion of the new subsistence economy was dictated by differences in growth rates (demic diffusion) or intergroup cultural transmission (cultural diffusion) (7, 8, 14).

The spread of agriculture was also characterized by the transmission of neutral cultural traits, such as pottery decoration styles, that were transmitted along with selectively advantageous traits leading to a statistical association between neutral and adaptive traits. This process is often referred to as cultural hitchhiking [*sensu* (15)], and quantitative models of such a phenomenon, or more broadly cultural linkage, have been examined in the past (15–18). For example, Ackland et al. (15) argue that in the presence of a heterogeneous landscape, we should expect to observe a diffusion boundary where the wave of demographic advance is temporarily halted and adaptive trait variants are transmitted to incumbent communities without the hitchhiking cultural variants, leading to the emergence of a permanent “cultural boundary.” While processes leading to an association between different cultural traits can vary, from simple functional or cognitive associations to the consequences of certain types of transmission bias (e.g. prestige-biased cultural transmission leading to the adoption of multiple independent traits possessed by the same prestigious individual), the extent by which neutral and adaptive traits can be statistically associated as a consequence of intergroup exogamy remains virtually unexplored.

Here, via an individual-based simulation, we examine how the link between variants of adaptive and neutral traits is maintained or lost due to different postmarital residence rules and transmission pathways within a two-population model. Our objective is to provide potential insights into the baseline conditions that can promote or hinder cultural hitchhiking during the spread of beneficial traits between initially distinct human groups. The analyses we undertake extend from previous models of cultural hitchhiking. For example, Ackland et al. (15) assume that intergroup transmission occurs only for adaptive traits, while here, we allow both neutral and adaptive traits to be transmitted. Our simulation exercise also differs from other demic-cultural diffusion models (e.g. ref. 8), where neither the interplay between postmarital residence rules and transmission pathways nor the statistical association between neutral and adaptive traits are explored. Our model does present some similarities to cultural evolutionary models on ethnic markers (e.g., refs. 19 and 20). However, we note that in our study, neutral traits are entirely nonfunctional, have no signaling value, and dynamics are not driven by cooperation/coordination games. In particular, as noted in the model description below, we assume that the association between the adaptive and neutral traits is not the result of the latter promoting indirectly cooperative/coordinating tasks (i.e. acting as a marker trait) but purely the consequences of different patterns of postmarital migration rules and transmission rules.

## The Model

**Fig. 1.**
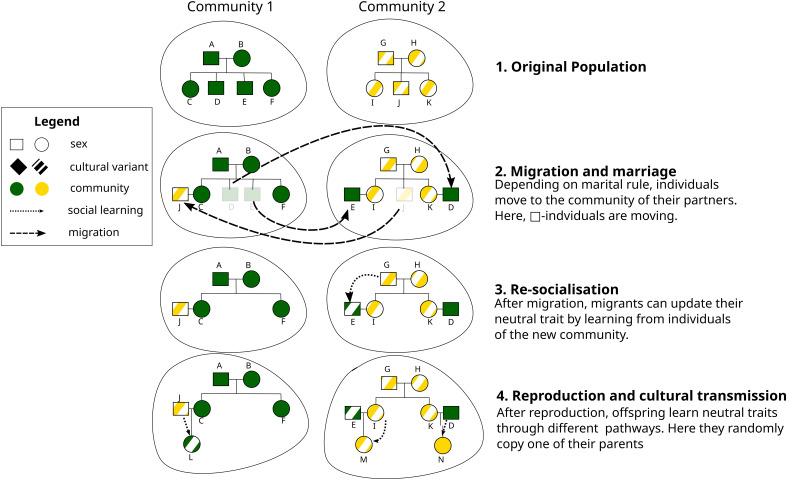
Generalized schematic of the model with two communities (1). In (2), individuals of the same sex D, E, and J migrate and find a partner in their new communities. In (3), individual E engages into resocialization via oblique cultural transmission. In the last step (4), the new pairs reproduce giving birth to individuals L, M, and N who adopt the cultural variants of one of the parents via vertical transmission.

We consider a population of individuals distributed across several communities, *j*, located at positions $\left(x_{j},y_{j}\right)$ in a two-dimensional 10×10 grid. Each community consists of individuals with the following state variables: *sex*, *age*, and the adopted variants of *z* neutral traits, $c_{1},c_{2},\ldots ,c_{z}$ as well as the variants of three adaptive traits, $a_{1},a_{2},a_{3}$. Fig. 1 shows a generalized schematic of the model highlighting its core processes, involving postmarital movement (step 2) and up to two episodes of cultural transmission, namely vertical transmission in the community of origin (step 4) and resocialization after postmarital migration (step 3).

### Cultural Traits.

We consider *z* cultural traits, $c_{1},c_{2},\ldots ,c_{z}$, which do not affect the survival or reproductive capacity of the individuals or the traits themselves, i.e. they are selectively neutral. Each trait can assume two distinct variants, i.e. $c_{i}\in \left\{0,1\right\},\;i=1,\ldots ,z$. We will refer to these as 0-variant and 1-variant. As detailed in *Cultural Transmission of Neutral Traits*, traits differ in their transmission pathways.

Further, we consider three adaptive traits, $a_{1},a_{2},a_{3}$. Again, each trait can assume two variants, i.e. $a_{i}\in \left\{0,1\right\},$i=1,2,3. These variants model the absence or presence of three different components of a novel beneficial technology. The presence of each component provides an individual with a fitness benefit, $f_{1},f_{2}$ and *f*_3_, respectively, resulting in an increased reproductive capacity (*Aging, Death, and Reproduction*). In contrast to the neutral traits, the adaptive traits $a_{1},a_{2}$ and *a*_3_ are defined at the community level, i.e. all individuals within a community have the same trait variant. This is based on the assumption that the new technology cannot be adopted and performed by a single person but only by the community as a whole.

### Demographic Processes.

#### Aging, death, and reproduction.

At each time step, individuals age and can die with an age-dependent probability *p*_death_. We assume sexual reproduction, i.e. only pairs of individuals (see *Marriage and Migration* for how they are formed) where both partners are within the age interval [18,45] can produce one offspring per time step. Parents and all their offspring are considered a family. Reproduction occurs with a probability $b+f_{1}1_{a_{1}=1}+f_{2}1_{a_{2}=1}+f_{3}1_{a_{3}=1}$, where *b* describes the baseline reproductive rate and the sum $f_{1}1_{a_{1}=1}+f_{2}1_{a_{2}=1}+f_{3}1_{a_{3}=1}$ captures the increase of this baseline rate due to the potential adoption of more beneficial technology. The function $1_{a_{i}=1}$ describes the indicator function, which is 1 if the condition $a_{i}=1$ is fulfilled and 0 otherwise. Offspring are assigned random sex and engage in cultural transmission to obtain the variants of their neutral cultural traits (*Cultural Transmission of Neutral Traits*). They have the same variants of the adaptive cultural traits as their community.

#### Marriage and migration.

At each time step, all unmarried individuals older than 18 have an opportunity to marry someone from a different community. This is achieved by adding all unmarried individuals to a common pool and subsequently creating random pairs of opposite sex and different communities of origin. If an individual is not paired, they return to their community and participate in the same routine in the subsequent time step. As both partners are chosen from different communities, pairs must decide where to reside together. This decision is controlled by the parameter *p*_location_, which describes the probability that the pair moves to the community of the female individual. We examine two scenarios:

- Bilocality ($p_{\text{location}}=0.5$): The location of the pair is chosen at random—on average, half of all pairs reside in the community of the female individual while the other half resides in the community of the male individual.
- Patrilocality ($p_{\text{location}}=0$): The pair always resides in the community of the male individual.

Matrilocality ($p_{\text{location}}=1$) is not considered as it provides in our model similar results to patrilocality but with opposite *p*_bias_-values (see below). All pairs stay together until one individual dies, with no opportunities for remarriage.

#### Community fission.

As we allow for temporally changing population sizes, we assume that if community *j* reaches size *n*_fission_, it fissions into two communities. To do so, we split the original community by assigning each family of *j* to one of the two new communities at random; while trying to ensure that both new communities are of similar size. The larger community occupies the original location $\left(x_{j},y_{j}\right)$ while the other fills a random empty location. If all cells are occupied, the simulation stops.

### Cultural Transmission of Neutral Traits.

We assume that cultural transmission can only happen at two points in time (Fig. 1)—first at birth and second after migration to a new community. At birth, transmission occurs vertically (*v*). Transmission after postmarital migration occurs horizontally or obliquely with probability *p*_transmit_. More formally, following ref. 1, vertical transmission is defined as transmission from the (genetic) parents; horizontal transmission is defined as transmission from peers, i.e, from individuals of the same generation, described here as all individuals with an absolute age difference below 20 y; and oblique transmission is defined as transmission from individuals who are at least 20 y older.

We assume each cultural trait considered has a different combination of transmission pathways before and after marriage (Table 1), with a total of five unique combinations. Additionally, we consider the effects of a sex bias, modeled as the preference for learning from one sex, for all transmission pathways. More specifically, individuals will select with probability *p*_bias_ a female role model. Thus, when $p_{\text{bias}}=0.5$ we have no sex bias, i.e. individuals are equally likely to learn from male and female individuals. In contrast, when $p_{\text{bias}}=0$ or $p_{\text{bias}}=1$, males only learn from males (i.e. the cultural trait is patrilineal as only transmitted through the male line) and females learn only from females (i.e. the cultural trait is matrilineal as only transmitted through the female line), respectively. Put differently, for $p_{\text{bias}}=0$ and $p_{\text{bias}}=1$, the variants from individuals of the opposite sex are never transmitted, and we analyze only the variants carried by individuals of the sex toward which the bias is directed. We note that if the focal individual does not find an individual to learn from, it retains the cultural variant learned in its community of origin via vertical transmission.

**Table 1.** Transmission pathways for each of the 5 cultural traits after postmarital migration

Trait	*p*_transmit_	Pathway
*c*_1_	0	–
*c*_2_	0.9	horizontal
*c*_3_	1	horizontal
*c*_4_	0.9	oblique
*c*_5_	1	oblique

### Adoption of Adaptive Traits.

In contrast to neutral traits, adaptive traits are considered as community-level traits, i.e. all individuals in the community have the same variant. Transmission of the adaptive traits, $a_{1},a_{2},a_{3}$ occurs independently and after migration events at each time step. As the traits *a*_*i*_ are community-level traits we refer to their transmission as adoption in the following to avoid confusion with the transmission of neutral traits.

The probability $p_{a_{i}}$ of the community changing the variant of the trait *a*_*i*_ is given by

[1] $$p_{a_{i}}=\frac{k_{i}^{1-\beta }}{k_{i}^{1-\beta }+{\left(n-k_{i}\right)}^{1-\beta }},$$

where *n* is the size of the community (including the migrants), and *k*_*i*_ is the number of migrants possessing the variant of *a*_*i*_ that is different from the one currently adopted by the focal community. The parameter *β* controls the likelihood of a successful adoption of the novel variant carried by migrants. When *β* = 0, the probability of adopting the novel variant equals the proportion of migrants with such variant in the community. However, negative values of *β* decrease $p_{a_{i}}$. For large negative values, the probability of adoption becomes extremely low, almost irrespective of the number of migrants moving in with the novel variant. Conversely, positive values of *β* will increase the probability of adopting the novel variant, even when the number of migrants bringing such a variant is comparatively small (*SI Appendix*, Figs. S3 and S4).

## Experimental Design

We initialize each simulation with eight “incumbent” and two “migrant” communities of size 70. The former possess the trait variants $a_{i}=0$, i=1,2,3 and $c_{i}=0$, i=1,…,z, whereas the latter possess new variants, i.e. $a_{i}=1$, i=1,2,3 and $c_{i}=1$, i=1,…,z. For simplicity, we will refer to communities with the adaptive variants of the incumbent population (i.e. with $a_{1}=a_{2}=a_{3}=0$) as type A communities. Conversely, we will refer to communities with the adaptive variants of the migrant population (i.e. with $a_{1}=a_{2}=a_{3}=1$) as type B. The remaining communities, i.e., those partially adopting the adaptive technology, will be referred to as type C communities.

At initialization, sex and age of all individuals are chosen at random. We then ran 500 time steps to allow all communities to reach a stable age structure. During this burn-in phase, we did not allow any form of cultural transmission, adoption, or postmarital migration, while newly created communities following fission events were removed from the system to maintain the initial number of type A and type B communities.

We conducted our simulation experiment using fixed values for *p*_death_= 0.15 (0 to 5 y old), 0.01 (6 to 40 y old), 0.02 (41 to 65 y old), 0.05 (66 to 85 y old), 1 (>85 y old) [loosely based on ethnographically observed figures for hunter-gatherer societies (21)]; *b* = 0.3224 (resulting in stable populations with effective growth rates as shown in *SI Appendix*, Figs. S1 and S2), and $n_{\text{fission}}=100$. Multiple values were used for the remaining parameters (Table 2) to explore a wide range of experimental scenarios. For each parameter combination, we ran 125 repetitions, which were stopped once the 10×10 grid was filled.

**Table 2.** Sweeping parameter ranges for the simulation experiments

Parameter	Description	Range
*β*	Learning bias for adopting novel variants from migrants.	−10, 0
*f*_1_, *f*_2_, *f*_3_	Reproductive bonus of adaptive trait variants.	0, 0.005, 0.015
*p*_transmit_	Probability of postmarital social learning.	0, 0.9, 1
*p*_bias_	Sexual bias in social learning (i.e. probability of learning from a female individual)	0, 0.5, 1
*p*_location_	Probability of postmarital residence at female partner	0 (patrilocality), 0.5 (bilocality)

Our experiment design is set to explore the specific conditions under which the association between adaptive and neutral variants observed initially in the type B communities persists over time. More specifically, we explore how potential differences in growth rates between populations (i.e. different values for *f*_1_, *f*_2_, and *f*_3_) and the degree by which adaptive traits can be adopted (i.e. different values of *β*) condition the joint spread (or lack thereof) of the variants of the neutral and adaptive traits of the migrant population ($a_{i}=1$, i=1,…,3 and $c_{i}=1$, i=1,…,z). For that, we consider three values for the growth benefits *f*_*i*_ characterizing situations of no growth advantage ($f_{i}=0$, i.e. no difference in the growth rate between both community types), small growth advantage ($f_{i}=0.005$, i.e. the effective growth rate of type B communities is slightly higher than of type A communities and high growth advantage ($f_{i}=0.015$, i.e. the effective growth rate of type B communities has almost doubled—it increased to roughly 0.015 compared to roughly 0.008 for type A communities (*SI Appendix*, Fig. S1, *Left*). For the strength of adoption of the adaptive traits *a*_*i*_, we consider two values. While β=−10 describes the situation of no adoption—Eq. **1** equals almost 0 for all values of numbers of migrants and community sizes—, *β* = 0 describes the situation of relatively strong adoption. *SI Appendix*, Fig. S4 shows that even for one migrant the adoption probability (per time step) has a value of roughly 0.01. Further, we explore how different transmission pathways (Table 1), biases (controlled by *p*_bias_), and postmarital residence rules (controlled by *p*_location_) interact with the joint spread process.

To do so, we quantify the strength of the association between variants of the adaptive and neutral trait by measuring how the spread of the adaptive variant is mirrored by the spread of the neutral variant. More precisely, we calculate the proportion of the individuals in type B and C communities, i.e. in communities that have adopted the new technology to some degree, that possess $c_{i}=1$.

## Results

Given the past focus on demic and cultural diffusion as contrasting, but not mutually exclusive, explanations for phenomena like the spread of farming, we structured our analysis as follows. First, we consider a scenario that effectively, from the standpoint of cultural traits, resembles a pure demic diffusion. More specifically, we assume different growth benefits, $f_{i},\;i=1,2,3$ derived from the trait variants $a_{i}=1,\;i=1,2,3$ but do not allow for the adoption of those variants between communities, i.e. we set β=−10. Further, we do not allow for individual-level cultural or demographic processes apart from vertical transmission of variant values at birth. In particular, we do not allow for “culturally relevant” migration between communities, i.e. migration that can introduce locally novel trait variants, which may subsequently be transmitted between individuals of this community, and different forms of resocialization after migration. We analyze the effects of different growth benefits, *f*_*i*_ on: i) the spread of the adaptive trait variants $a_{i}=1$, ii) the strength of the association between the neutral and adaptive traits, i.e. the signature of hitchhiking, and iii) the level of cultural diversity of neutral traits in the population. We then generalize the very restrictive assumptions of demic diffusion by exploring the effects of the interplay between different forms of culturally relevant migration (caused by intergroup exogamy), of resocialization, and different values of *f*_*i*_ on i), ii), and iii).

Second, we consider a scenario resembling pure cultural diffusion. Again, here, we only consider the implications from the standpoint of the cultural traits and do not consider genetic admixture. We assume that the community-level trait variants $a_{i}=1,\;i=1,2,3$ do not confer a growth advantage to the adopting community, i.e. $f_{i}=0$, but allow for the adoption of these traits between communities, i.e. *β* = 0. As above, we assume the absence of any individual-level cultural or demographic processes apart from the vertical transmission of cultural variants at birth. We analyze the effect of different adoption strengths *β* on i), ii), and iii). Again, we generalize in the next step the very restrictive assumptions of cultural diffusion by exploring the effects of the interplay between different forms of culturally relevant migration, of resocialization, and different values of *β* on i), ii), and iii).

Third, we analyze the combined effects of demic and cultural diffusion. We assume that the community-level trait variants $a_{i}=1,\;i=1,2,3$ confer a growth advantage $f_{i}>0$, i=1,2,3 to the adopting community and allow for the adoption of these traits between communities, i.e. *β* = 0 and analyze their effects on i), ii), and iii) conditioned on different assumptions about culturally relevant migration and resocialization.

We focus our discussion on the results under patrilocality, given that key features were observed across both postmarital residence rules. *Bilocality* briefly summarizes unique differences observed in the simulation output when both male and female individuals relocate after marriage.

### Influence of Growth Benefits *f*_*i*_ without the Adoption of *a*_*i*_ Traits (*f*_*i*_ > 0, *β* = −10).

**Fig. 2.**
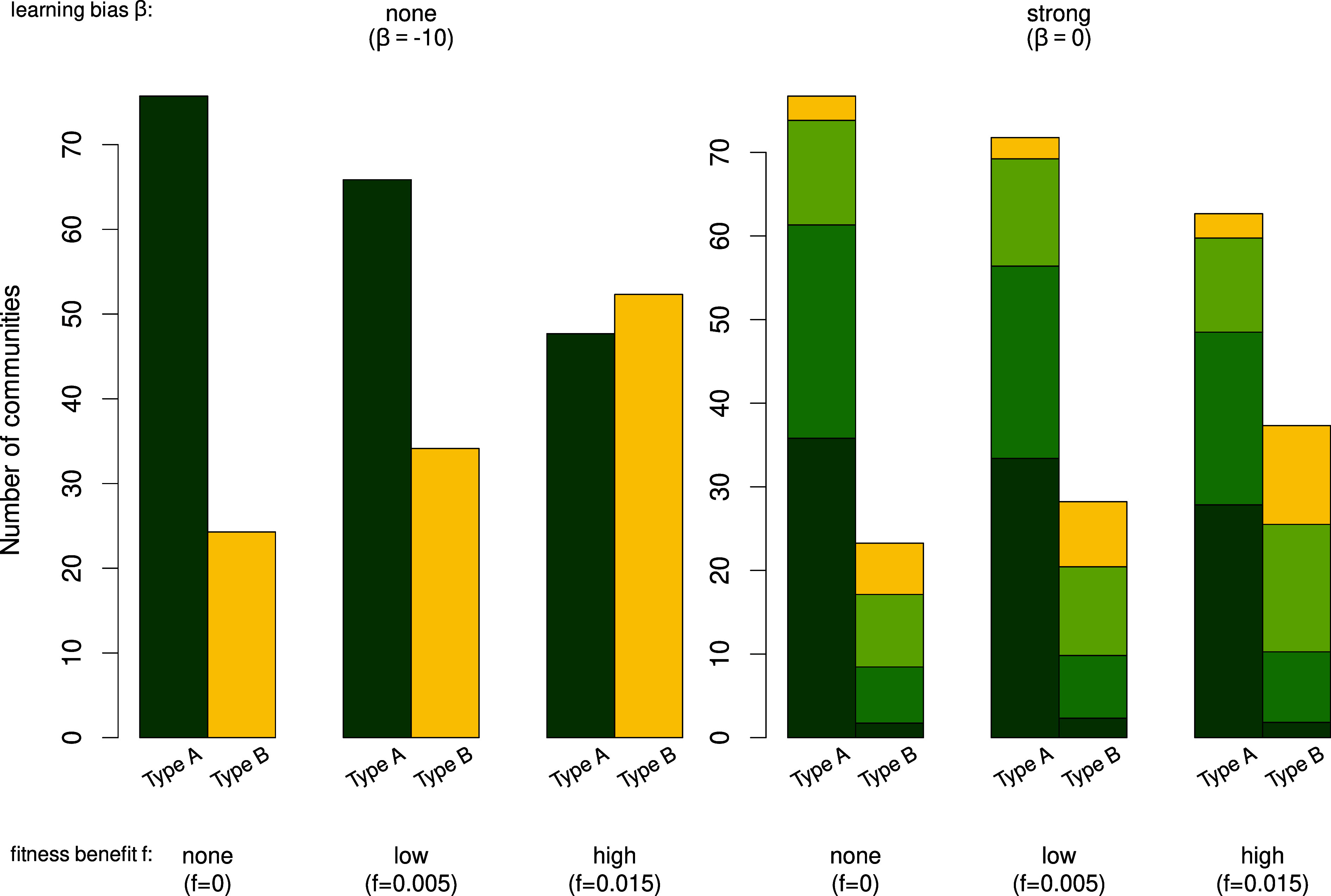
Average growth behavior at the end of the simulation of the number of communities of types A (dark green), B (yellow), and C (green and light green) conditioned on whether they descend from the initial eight type A or two type B communities for $f=f_{1}=f_{2}=f_{3}=0,0.005;\;0.015$ and β=−10;0.

We start by analyzing the spread behavior of the novel technology, i.e. of the community-level adaptive traits *a*_*i*_, i=1,2,3. Fig. 2 shows the average number of communities of type A, B, and C at the end of the simulation, conditioned on whether they descended from the initial eight type A and two type B communities, respectively, (for different values of *f*_*i*_ and *β*). The *Left* panel for β=−10 shows that—as expected—higher growth benefits *f*_*i*_ derived from $a_{i}=1$ result in higher average numbers of type B communities. We note that due to the absence of the adoption of adaptive traits *a*_*i*_ no community of type C emerges. Put differently, when $f_{i}>0$, individuals of type B communities reproduce more frequently, leading to a higher fission rate, which ultimately results in a higher proportion of the adaptive trait variants $a_{i}=1$ in the population. Because reproductive rates are dependent on *f*_*i*_-values, higher values of *f*_*i*_ lead to a higher proportion of the novel technology in the population.

#### “Pure” demic diffusion (*f*_*i*_ >0, *β* = −10, *p*_bias_ = 0).

When $p_{\text{bias}}=0$, the focal trait is only transmitted between males; as a result, effectively, there is no cultural exchange between different communities given our patrilocal settings. In other words, migration is not culturally relevant. While we are still in the presence of genetic admixture between populations, the lack of cultural exchange between communities makes this, de facto, an entirely demic diffusion process.

**Fig. 3.**
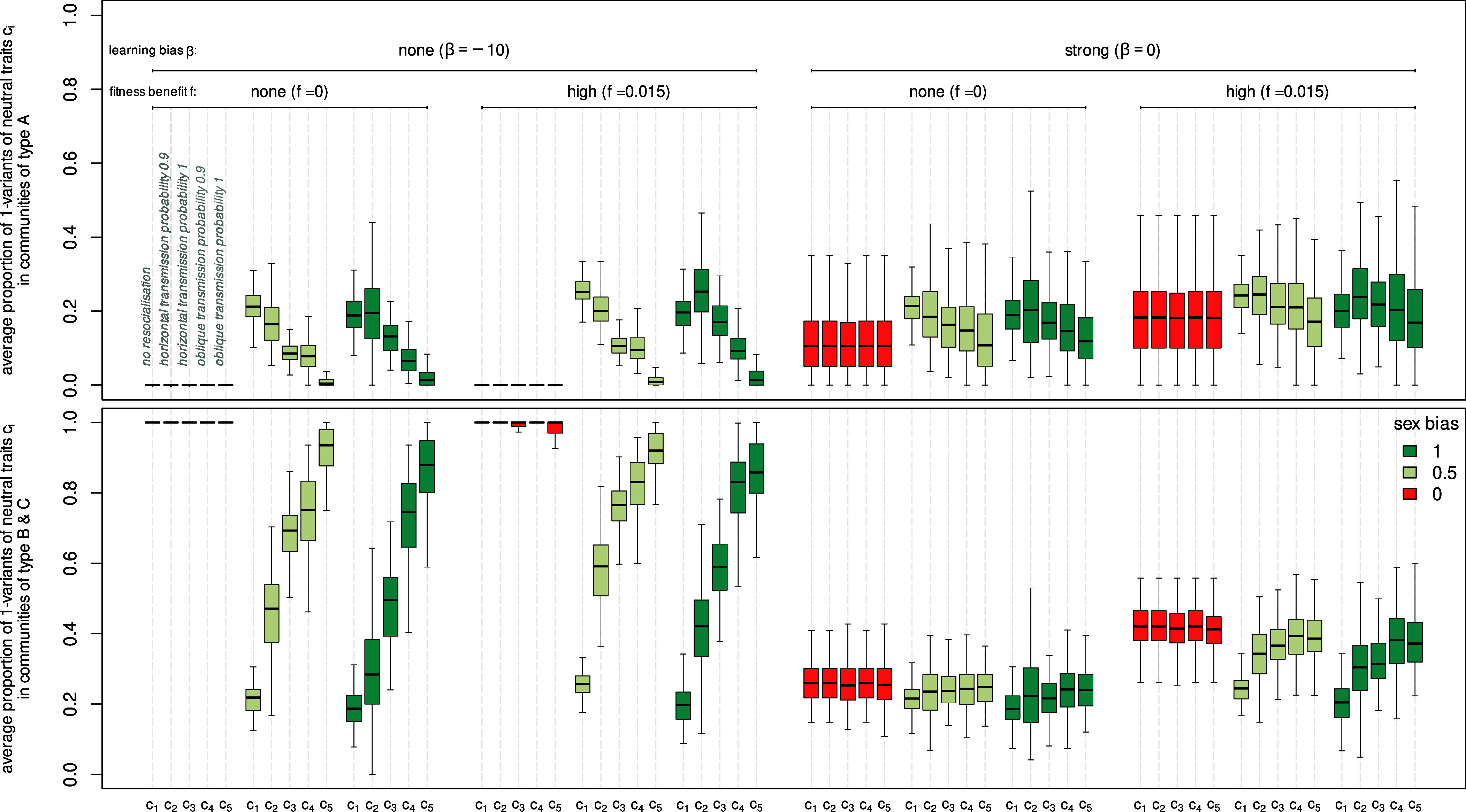
Proportion of individuals in type A communities that possess $c_{i}=1$, i=1,…,5 (*Top* row), proportion of individuals in type B and C communities that possess $c_{i}=1$, i=1,…,5 (*Bottom* row) at the end of each simulation for $f=f_{1}=f_{2}=f_{3}=0.005;\;0.015$, β=−10;0, different transmission pathway labeled by $c_{1},\ldots ,c_{5}$ (Table 1) and $p_{\text{bias}}=0$ (color red), $p_{\text{bias}}=0.5$ (color gray), $p_{\text{bias}}=1$ (color blue).

**Fig. 4.**
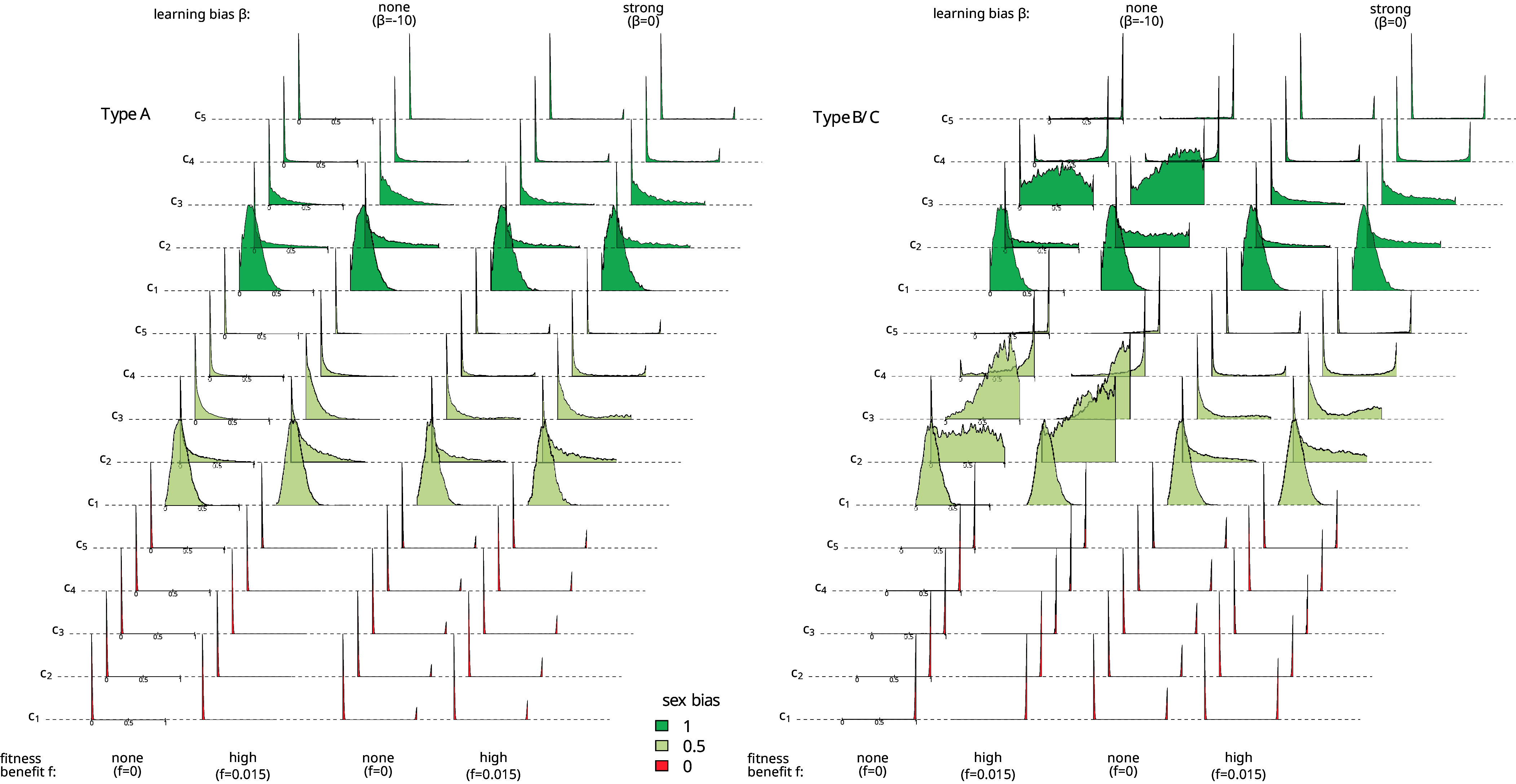
Distributions of the proportion of individuals carrying $c_{i}=1$ in type A (*Left* panel) and type B/C (*Right* panel) communities at the end of the simulation accumulated over 125 repetitions for β=−10 and $f_{i}=0;0.015$, i=1,2,3 (first and second columns), *β* = 0 and $f_{i}=0;0.015$, i=1,2,3 (third and fourth columns), labeled by different transmission pathways (Table 1) and $p_{\text{bias}}=0$ (color red), $p_{\text{bias}}=0.5$ (color gray), $p_{\text{bias}}=1$ (color blue).

To explore the association between neutral and adaptive trait variants, we record in Fig. 3 the proportion of neutral trait variants $c_{i}=1$, i=1,…,5 in type A (*Upper* row) and type B/C (*Bottom* row) communities (for different values of *p*_bias_, *f*_*i*_, and *β*). As mentioned in *Cultural Transmission of Neutral Traits*, when $p_{\text{bias}}=0$ (and similarly when $p_{\text{bias}}=1$), cultural variants are exclusively transmitted between male or female individuals, respectively. Hence, in Fig. 3, we show only the proportions of the cultural variants among one sex under these settings. To explore the level of cultural diversity of neutral traits, Fig. 4 shows the distribution of the proportion of individuals carrying $c_{i}=1,\;i=1,\ldots ,5$ in type A (*Left*) and type B/C (*Right*) communities at the end of the simulation accumulated over 125 simulations (for different values of *p*_bias_, *f*_*i*_, and *β*).

Fig. 3 (color red, β=−10, $f_{i}=0;0.015$, four sets of five boxes and whiskers) show the results for the 5 transmission pathways $c_{1},\ldots ,c_{5}$ (Table 1). Unsurprisingly, all transmission pathways result in similar proportions, as effectively, no cultural exchange between communities occurs despite the genetic admixture. As expected, all type A communities retain the 0-variant of the neutral traits and all type B communities the 1-variant of the neutral traits, regardless of the value of *f*_*i*_. Consequently, all communities are culturally homogeneous (see Fig. 4, first two columns on each panel, red distributions). As a result, we observe the strongest possible signature of hitchhiking, where the adaptive variants $a_{i}=1$, i=1,2,3 are always associated with all neutral variants $c_{i}=1$, i=1,…,5.

In the following, we examine more closely the interactions between different values of *f*_*i*_, transmission pathways $c_{1},\ldots ,c_{5}$ and values of the sex bias, *p*_bias_ and their consequences on the signature of hitchhiking and the level of cultural diversity in the population. We note that the spread behavior of the community-level adaptive traits *a*_*i*_ is unaffected by different transmission pathways and sex biases.

#### Culturally relevant migration and no resocialization (*f*_*i*_ > 0, *β* = −10, *p*_bias_ > 0, *c*_1_).

When $p_{\text{bias}}=1$, the transmission of cultural traits occurs only between female individuals. Furthermore, for the trait *c*_1_, this transmission process occurs only within the community where individuals are born as—given our patrilocal setting—we assume no postmarital resocialization, $p_{\text{transmit}}=0$.

Fig. 4 (second column of each panel, blue distributions for *c*_1_) shows that frequent migration has the same effect on cultural diversity between communities as it has on genetic diversity. We observe that cultural diversity with respect to *c*_1_ is homogenized across all communities—the median proportions of 1-variants for type A and B communities are equal and relatively low (see Fig. 3, color blue, first box, and whisker, β=−10, $f_{i}=0;0.015$) implying that we observe a disruption of the hitchhiking signature. Further, the median proportions of individuals carrying $c_{1}=1$ in type B communities are only weakly influenced by different *f*_*i*_-values. Due to the initial condition of our simulation, where eight communities are of type A and two of type B, we observe a frequent migration of individuals of type A communities into type B communities at the beginning, consequently many $c_{1}=0$ variants benefit from the higher growth rates of the type B communities, and as a result, homogenization occurs relatively fast.

The low association between the adaptive and neutral traits in the absence of resocialization is observed even when $p_{\text{bias}}=0.5$, i.e. when the transmission of the neutral traits is not conditioned by the sex of the individuals. Furthermore, in the absence of resocialization and without a sex-biased transmission, higher fitness in the adaptive traits seems to lead to a marginal increase in the proportions of type-1 variants across all populations.

#### Culturally relevant migration and resocialization (*f*_*i*_ > 0, *β* = −10, *p*_bias_ > 0, *c*_2_, …, *c*_5_).

Again, migration and transmission happen only among female individuals, but, in contrast to the above settings, migrants have an opportunity to resocialize, either via horizontal transmission with $p_{\text{transmit}}=0.9;\;1$ (*c*_2_, *c*_3_) or oblique transmission with $p_{\text{transmit}}=0.9;\;1$ (*c*_4_, *c*_5_).

##### Oblique transmission.

Transmission can only occur from individuals who are at least 20 y older than the focal migrant. Given that all female individuals migrate after they reach maturity, the pool from which they can learn after relocating effectively consists of other female individuals who have not recently migrated to the community.

We examine first the results when $p_{\text{bias}}=1$. In Fig. 4 (second column in both panels, blue distributions for *c*_4_,*c*_5_), we observe that oblique transmission counteracts the effect of migration. When $p_{\text{transmit}}=1$, we observe strong cultural homogeneity; every migrant learns the variant of *c*_5_ from the older generations in the new community, leading to culturally homogeneous communities where type A communities are characterized by $c_{5}=0$ and type B communities by $c_{5}=1$. This is in stark contrast to the pattern observed for *c*_1_, i.e. without resocialization, and therefore closely resembles a pure demic diffusion discussed above (see also Fig. 3, color blue, fourth and fifth box and whisker, β=−10, $f_{i}=0.015$), resulting in a strong signature of hitchhiking. We note that the proportions shown in Fig. 3 are not precisely at 0 and 1, respectively. This is caused by the fact that in our simulations, there are rare circumstances where there are no older female individuals to learn from. In this case, the migrant keeps its original variant and does not engage in resocialization. Cultural homogeneity is slightly reduced when the frequency of resocialization is decreased to *p*_transmit=0.9_ (*c*_4_), but the generally high levels of hitchhiking are still observed.

*SI Appendix*, Fig. S7 also shows that the influence of different values of *f*_*i*_ on the effects of oblique transmission is relatively low, with slightly higher proportions of 1-variants for higher values of *f*_*i*_.

When $p_{\text{bias}}=0.5$, we observe similar median proportions of individuals with $c_{i}=1$, i=4,5 in type A and type B communities as irrespective of whether migrants learn from an older male or female individual, they, theoretically, carry the local trait variant. The distribution of 1-variant across the two types of communities is broadly similar to those observed under $p_{\text{bias}}=1$, although with higher levels of skewness, especially for *c*_4_, indicating a comparatively larger number of communities where the proportions differ from 0 or 1 due to the rare circumstances where there are no older individuals to learn from.

##### Horizontal transmission.

Transmission can only occur from individuals who are in the same generation as the migrant. Importantly, this means that migrants could learn from other migrants, who are, by definition, from the same generation. Consequently, horizontal transmission—in this form—does not lead to the same levels of cultural homogeneity within communities as oblique transmission; in fact, it does the opposite, especially for type B communities. Fig. 4 (second columns on both panels, blue distributions for *c*_2_,*c*_3_) shows that the distributions cover the entire interval [0,1] whereby type B communities are more likely to possess higher proportions of individuals with 1-variants compared to type A communities (see also Fig. 3, color blue, second and third box and whisker, β=−10, $f_{i}=0.015$).

Further, *c*_2_ and *c*_3_ traits have considerably lower median proportions of 1-variants among type B communities, which is indicative of a weaker signature of hitchhiking compared to oblique transmission (*c*_4_ and *c*_5_). Again, the influence of different values of *f*_*i*_ on the effects of horizontal transmission is relatively low, with slightly higher proportions of 1-variants with higher values of *f*_*i*_ (*SI Appendix*, Fig. S7).

For $p_{\text{bias}}=0.5$, the median proportion of individuals with $c_{i}=1$ is lower for type A communities and higher for type B communities for horizontal transmission, i.e., *c*_2_ and *c*_3_, (see Fig. 3, color gray, second and third box and whisker, β=−10, $f_{i}=0;0.015$) due to the fact that now, in the assumed patrilocal setting, at least half of the time, migrants copy the local variant from male individuals.

Summarizing, in the absence of the adoption of the technological traits *a*_*i*_, a novel variant can only spread within the population if it provides a (high) growth advantage to its adopters. In accordance with previous results (cf. 15), we observe a strong signature of hitchhiking under pure demic diffusion. However, this signature is eradicated if we allow e.g. for culturally relevant migration of individuals caused by intergroup exogamy (i.e. situation with $f_{i}>0$, β=−10, $p_{\text{bias}}>0$, and *c*_1_), irrespective of the value of *f*_*i*_. Resocialization in the form of oblique transmission preserves the signature of hitchhiking, even in the face of (culturally relevant) migration.

### Influence of the Adoption of *a*_*i*_ Traits without Growth Benefits (*f*_i_ = 0, *β* = 0).

Keeping the last section’s structure, we first analyze how the novel technology gets adopted. Fig. 2 (columns for $f_{i}=0$) shows that—as expected—the number of communities descended from the initial type A and B communities, respectively, keep a similar 80:20 ratio over time but, due to the possibility of transmitting traits $a_{i},\;i=1,2,3$, the descendants can be of any type. In particular, we observe the emergence of type C communities, i.e communities characterized by the partial adoption of the novel technology. Interestingly, this means that when the adoption of traits *a*_*i*_ is possible, most communities adopt parts of the novel technology, even if it does not provide a growth advantage. It holds that the higher the strength of cultural transmission, i.e. the larger the *β*-value, the more pronounced this pattern is.

#### “Pure” cultural diffusion (*f*_i_ = 0, *β* = 0, *p*_bias_ = 0).

For $p_{\text{bias}}=0$, Fig. 3 (color red, *β* = 0, $f_{i}=0$, four sets of five boxes and whiskers) shows the results for the five transmission pathways $c_{1},\ldots ,c_{5}$ for male individuals. As in the section above, all transmission pathways result in similar proportions, as effectively, no cultural exchange between communities occurs. However, we see a drastic reduction in the proportion of individuals with type 1-variants in type B/C communities compared to when β=−10. This is caused by the fact that most type C communities descend from the initial type A communities, which are characterized by $c_{i}=0$. Fig. 4 (third column on both panels, red distributions) shows that all communities are culturally homogeneous, i.e. all individuals within a community possess either $c_{i}=0$ or $c_{i}=1$ now there are communities of type A that possess $c_{i}=1$ and communities of type B $c_{i}=0$. Adoption of the traits *a*_*i*_ of strength *β* = 0 (see *SI Appendix*, Fig. S4 for an exploration of the probability with which communities change their *a*_*i*_-variants for different *β*-values) leads to a disruption of the signature of hitchhiking. It holds, the more likely the adoption of the traits *a*_*i*_, the weaker the signature.

As above, we examine now more closely the interactions between the adoption of traits *a*_*i*_, transmission pathways $c_{1},\ldots ,c_{5}$ and values of the sex bias, *p*_bias_ and their consequences on the signature of hitchhiking and the level of cultural diversity in the population. We note again that the spread behavior of the community-level adaptive traits *a*_*i*_ is unaffected by different transmission pathways and sex biases.

#### Culturally relevant migration and no resocialization (*f*_i_ = 0, *β* = 0, *p*_bias_ = 1, *c*_1_).

Fig. 4 (third column on both panels, blue distributions for *c*_1_) shows that cultural diversity with respect to *c*_1_ is homogenized across all communities leading to low equal median proportions of 1-variants for type A and B/C communities (see Fig. 3, color blue, first box and whisker, *β* = 0, $f_{i}=0$). Consequently, the hitchhiking signature is disrupted. Comparing the proportion of individuals carrying $c_{1}=1$ in type A and type B/C communities in Fig. 3 for beta=−10 and *β* = 0 we see that the adoption of traits *a*_*i*_ has almost no influence on the median proportion due to the fast homogenization process. With $p_{\text{bias}}=0.5$, we observe a very similar behavior.

#### Culturally relevant migration and resocialization (*f*_i_ = 0, *β* = 0, *p*_bias_ = 1, *c*_2_, …, *c*_5_).

Oblique and horizontal transmission follow the exact same dynamics as described in the relevant subsection above, but still, we see drastic differences in Fig. 3 between the median proportions of individuals carrying $c_{i}=1,\;i=2,\ldots ,5$ in type A and type B/C communities for $f_{i}=0$ and β=−10;0. The changes in median proportions for $p_{\text{bias}}=0$ under adoption of traits *a*_*i*_ provide us a useful frame of reference in this case. When $p_{\text{bias}}=0$, we observe the highest cultural differences between communities—the whole community has either adopted $c_{i}=0$ or $c_{i}=1$ [see Fig. 4 (third column on both panels, red distributions)] leading to the highest proportion of individuals with $c_{1}=1$ in type B/C communities. Culturally relevant migration and resocialization have the potential to alter this pattern. While perfect oblique transmission, i.e. *c*_5_, generates very similar results compared to $p_{\text{bias}}=0$; horizontal transmission and no resocialization, i.e. $p_{\text{transmit}}=0$, leads to more culturally heterogeneous communities. As a result, the median proportions of individuals with $c_{i}=1,\;i=2,\ldots ,5$ in type A and type B/C communities changes; however, due to the already low median proportion for $p_{\text{bias}}=0$, especially for type B/C communities, this change is much smaller compared to the setting with $f_{i}>0$ and β=−10 (Fig. 3). This greatly reduces the effect of the different transmission pathways on the hitchhiking signature—we observe a weak signature of hitchhiking for all considered transmission pathways. Furthermore, the higher the strength *β* of the adoption of traits *a*_*i*_, the weaker is the signature of hitchhiking for all transmission pathways considered (cf. also *SI Appendix*, Fig. S6).

With $p_{\text{bias}}=0.5$, we observe a very similar behavior.

Summarizing, in the absence of a growth benefit derived from adopting the community-level trait variants $a_{i}=1$, the adoption of traits *a*_*i*_ leads to the spread of parts of the novel technology through the population but not to the spread of the neutral trait variant $c_{i}=1$ associated with the new technology in the initial type B communities.

### Influence of the Adoption of *a*_*i*_ Traits with Growth Benefits (*f*_i_ > 0, *β* = 0).

Last, we analyze the situation where the novel technology is adaptive, i.e., the trait variants $a_{i}=1$ convey a growth benefit $f_{i}>0$ to their adopters, and can be transmitted between communities, i.e. *β* = 0.

Fig. 2 (columns for $f_{i}>0$, *β* = 0) shows that while the *f*_*i*_-values influence the number of communities descendent from the initial type A and B communities—the higher the *f*_*i*_-values, the more type B descendants—the *β*-values influence the type of the descendants. With higher values of *β*, more communities of type C exist. Similar to the situation with $f_{i}=0$ and *β* = 0, we see that most communities adopt parts of the novel technology and an increase in the *f*_*i*_-values results in a slight increase of type B communities.

When $p_{\text{bias}}=0$, Fig. 3 (color red, *β* = 0, $f_{i}=0,0.015$, four sets of five boxes and whiskers) shows the contrasting effects of increasing growth benefits *f*_*i*_ and increasing adoption strength *β*. While the median proportions of individuals carrying $c_{i}=1,\;i=2,\ldots ,5$, in type B/C communities are decreasing for higher values of *β*, positive values of *f*_*i*_ counteract this effect—the higher number of type C communities descended from the initial two type B communities (see Fig. 2, columns for $f_{i}>0$, *β* = 0)—and therefore strengthen the signature of hitchhiking.

When $p_{\text{bias}}=1$ and *c*_1_, Fig. 3 (color blue, first box and whisker, *β* = 0, $f_{i}=0.015$) shows again the same median proportions of individuals carrying $c_{1}=1$ in type A and type B/C communities. Compared to the situation with *β* = 0 and $f_{i}=0$ those proportions are slightly increased.

For $p_{\text{bias}}=1$ and $c_{i},\;i=2,\cdots ,5$, the impact of different pathways of resocialization is slightly more pronounced for higher *f*_*i*_-values [see Fig. 3 (color blue, second to fourth box and whisker, *β* = 0, $f_{i}=0.015$)], compared to the increased median proportions for $p_{\text{bias}}=0$.

Summarizing, the growth benefits, *f*_*i*_ of the adaptive trait variants $a_{i}=1$ and the strength *β* of the adoption of these adaptive traits *a*_*i*_ have opposing effects on the strength of the hitchhiking signature. While increasing the values of *β* results in weaker signatures, increasing the values of *f*_*i*_ results in stronger signatures. *SI Appendix*, Fig. S6 further reflects this point on the general diffusion of the 1-variant at the population level, showing the negative correlation between *β*- and *f*_*i*_-values.

### Bilocality.

Under bilocality (*SI Appendix*, Figs. S8 and S9 in supplementary material), our model settings lead to outcomes similar to patrilocality, with the highest level of hitchhiking observed in the presence of resocialization with oblique transmission. We note, however, that given both male and female individuals relocate after marriage, we no longer have a scenario resembling a “pure” demic diffusion (i.e. $f_{i}>0$, β=−10, $p_{\text{bias}}=0$). Thus, in the absence of cultural adoption, we no longer observe instances where type A community retains 0-variants, and type B community retains 1-variants. Instead, we observe a similar impact of the different transmission pathways across all settings of *p*_bias_.

Furthermore, we observe that when the adoption of the *a*_*i*_ variants is possible (i.e. *β* = 0), we observe lower retention of type-1 variants in the B/C type community under $p_{\text{bias}}=0$, indicating a lower level of hitchhiking promoted by the movement of both sexes.

## Discussion

The extent to which cultural traits are found in association is a key question in cultural evolution (17, 22–24). Mechanisms underlying such patterns can potentially vary, including cognitive associations, functional constraints, and shared transmission pathways, all leading to a systematic statistical association between two or more cultural traits. However, in some cases, such associations may specifically emerge between adaptive and neutral traits due to demic diffusion and intergroup migration.

Previous models have investigated such “cultural hitchhiking” (15), under the premise that the association between adaptive and neutral traits is maintained exclusively through demic diffusion. In other words, at the individual level, traits are jointly transmitted within communities; at the population level, communities grow in size and fission, spreading both adaptive and neutral traits in a given region. Under this model’s assumption, the community-level association between the two types of traits can be broken only if adaptive traits are transmitted between distinct populations. While the model by Ackland et al. (15) provides potential insights into how cultural boundaries of neutral traits can emerge during the wave of advance of a beneficial technology, the assumption that only demic diffusion can maintain the association between variants of adaptive and neutral traits is very restrictive. Here, we analyzed the extent to which other processes, such as individual migration via intergroup exogamy, can lead to cultural hitchhiking.

Our simulation explored the implications of the interaction between members of an incumbent and an immigrant population associated with novel, more beneficial variants of adaptive traits conveying reproductive advantage. We specifically considered the adoption of potentially adaptive technology and the transmission of neutral traits that require repeated interactions, achieved through the migration of individuals via intergroup exogamy. In our model, migrants may or may not acquire cultural traits via resocialization and become part of the learning pool for new individuals in the community. Our objective was to establish the specific conditions under which the statistical association between adaptive and neutral variants observed initially in the migrant communities persists over time. We analyzed the effect of different cultural and demographic processes, including a range of settings concerning the adoption of adaptive traits, the reproductive advantage gained by such traits, postmarital residence rules, and cultural transmission.

The results of our simulations suggest that cultural hitchhiking can be sustained via intergroup exogamy but only under a limited set of circumstances conditioned by postmarital residence rules and the nature of postmarital resocialization. First, we observe that hitchhiking can persist only under matrilocality or patrilocality but less so under bilocality. Under a sex-biased transmission regime, the nonrandom assortment of the sampling pool, i.e. the exclusive learning from one sex, has major implications on cultural hitchhiking. For example, traits associated exclusively with male individuals cannot be transmitted outside a community under patrilocality. That means an increase in the frequency of a particular cultural variant depends solely on whether communities with such variants have a competitive advantage in their growth rate (provided by the presence of a more beneficial adaptive trait). However, if traits are only transmitted from female individuals, the implications of a patrilocal postmarital residence rule change and depend on whether any resocialization occurs after postmarital migration.

Our results show for example, that if adaptive traits are not transmitted, high probability of resocialization can indeed sustain cultural hitchhiking. Our simulation suggests that while oblique transmission can sustain cultural hitchhiking, horizontal transmission can deteriorate its signal over time. This is due to the fact that traits can be transmitted between migrants, leading to dynamics not dissimilar to other studies exploring the interaction between migration and homophily (25).

Postmarital transmission pathway also has implications in terms of within-community vs. between-group cultural diversity. Under horizontal transmission, we observe the coexistence of both cultural variants within the same group, while oblique transmission keeps the between-community cultural diversity high and within-group cultural diversity to nil.

The simulation output provides an interpretative frame on possible mechanisms behind the spread of farming. Our results indicate that in order to sustain high levels of cultural hitchhiking, one of more of the following conditions should be met: 1) low probability of the adoption of the novel technology; 2) higher population growth rate offered by such novel cultural variant; and 3) high levels of cultural resocialization after postmarital migration of the exogamous sex. In the case of the spread of farming into Europe at least, we can presume that conditions 1) and 2) are likely to be present (26). There is also some limited archaeological evidence pointing to postmarital resocialization, Condition 3), in addition to ethnographic insights. Thus, individuals with very recent hunter-gatherer ancestry found in early farmer cemeteries in Central Europe are usually buried following the farmer burial rite with farmer material culture (27, 28). More generally, our model highlights the relevance of the role of migrants in the diffusion of novel cultural traits and how differences in cultural transmission practices after their relocation impact such a process. It thus provides some basic expectations on how sex-biased transmission and postmarital relocation can lead to different associations between functional and stylistic traits that can be further evaluated through ethnographic and anthropological field research.

Despite its complexity, our model is far from an exhaustive exploration of the interaction between incumbent and immigrating communities and their effects on the distribution of adaptive and neutral traits. For example, our model has assumed that adaptive traits are effectively transmitted without any bias, making the probability of incumbent communities adopting the more beneficial variant from the migrant communities the same as that of the migrant communities switching to the less beneficial variant of the incumbent groups. A less conservative scenario for example, one involving a payoff-biased transmission for the adaptive traits where the probability of adopting a more beneficial variant is higher, would have most likely led to a stronger hitchhiking signature. It is worth noting that several assumptions and model settings relevant specifically to the spread of agriculture may not apply to other scenarios. Our model assumes that migration between populations is the result of intergroup exogamy. We do not consider movements dictated by other factors that might be similarly sex-biased or entail migrations of entire families. One could envisage other contexts in which such an assumption, or the initial relative frequencies of the two populations, is different from the settings we explored. Further, we assumed that adaptive traits evolve at the group level to portray the nature of a beneficial trait that requires cooperative networks. We modeled the transmission of such adaptive traits *as if* communities were individuals, with a frequency-dependent probability of adoption. More complex models, for example, inspired by threshold models of collective behavior (29), could potentially reveal further insights into the coevolution of adaptive and neutral traits. For simplicity, here we explored a simpler scenario that nonetheless captures some specific aspects of subsistence strategies such as farming. We note that with our focus on understanding the spread of farming, the analysis of the first phase, i.e. before the population has reached its carrying capacity, is of particular interest. It is widely accepted that farmers usually migrate to new areas in small numbers and experience some sort of competitive growth advantage compared to the incumbent hunter-gatherer communities. If a hitchhiking signature has not been established in this first phase, it is unlikely to happen in later phases. Naturally, a precise analysis of later phases of the spread dynamic may involve the inclusion of population regulation which is outside the scope of this paper.

Finally, we note that despite the presence of rich ethnoarchaeological studies on the nature and implications of postmarital resocialization, there are virtually no formal theoretical, cultural evolutionary models building on that knowledge. As a result, on the one hand, cultural evolutionary models portraying the spread of adaptive and neutral traits neglect a potentially important factor driving some of the observed patterns in the archaeological record. On the other hand, the ethnoarchaeological literature is currently missing the opportunity to engage with formal models that can explicitly account for and compute the aggregate consequences of some of the most relevant ethnographically observed behavior on cultural transmission. We believe that processes such as postmarital residence and resocialization hold an important conceptual link for understanding the connection between individual-level cultural transmission and meta-population phenomena, providing a key bridge between cultural micro- and macroevolution.

## Supplementary Material

Appendix 01 (PDF)

## Data Availability

Source code and data have been deposited in Zenodo (30).
