# Supplementary material for: Postmarital residence rules and transmission pathways in cultural hitchhiking
Source: Proc Natl Acad Sci U S A. 2024 Nov 18;121(48):e2322888121. doi: 10.1073/pnas.2322888121 (PMC11621836; doi:10.1073/pnas.2322888121)
Supplement: Supplementary file 1 — Appendix 01 (PDF) [file pnas.2322888121.sapp.pdf]

Supporting Information for

Post-marital residence rules and transmission pathways in cultural hitchhiking

Simon Carrignon, Enrico R. Crema, Anne Kandler, Stephen Shennan

Stephen Shennan.
E-mail: s.shennan@ucl.ac.uk

This PDF file includes:

Supporting text
Figs. S1 to S9

Supporting Information Text

The source code and the data generated during simulations are available here: doi.org/10.5281/zenodo.12579204. The development version of the code is available here: github.com/simoncarrignon/mixed-transmission/

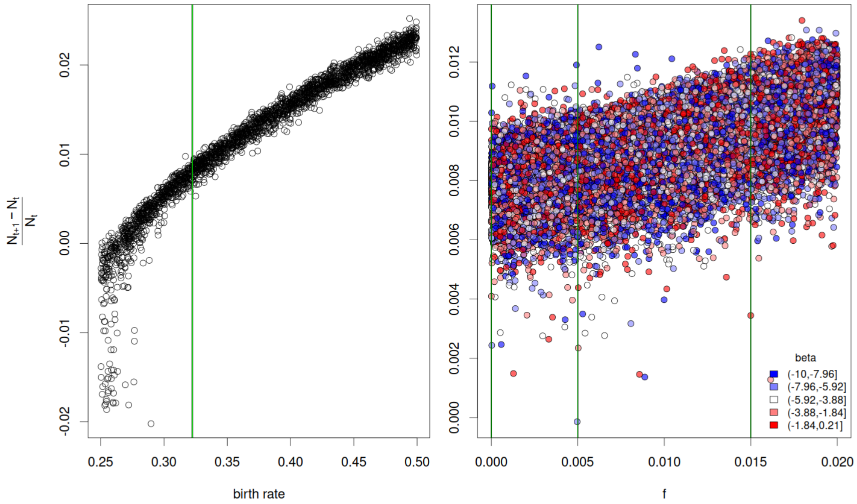

Fig. S1. Left: effective growth rate at the population level given base birth rate b. b has been randomly sampled between 0.20 & and 0.50, results for 2507 communities. No cultural transmission was allowed (i.e. $\beta = -10$) and no advantage was given to communities with adaptive traits (i.e. $f_i = 0$ so that the base rate is applied in the same way to all communities. Right: impact of f_i on the overall effective growth rate of the population for different values of β. Here, the overall growth advantage given to the community depends on how many adaptive variants $a_i = 1, i = 1, 2, 3$ the community possesses. The parameter values used and discussed in the paper are highlighted with vertical green lines, while the values found in the experiment under these parameters are represented in Figure S2. The base birth rate b used in the paper has been selected to avoid the potential extinction of the total population while keeping a population growth rate above 0.005 when no growth advantage was given to any community (i.e. when $f_i = 0$).

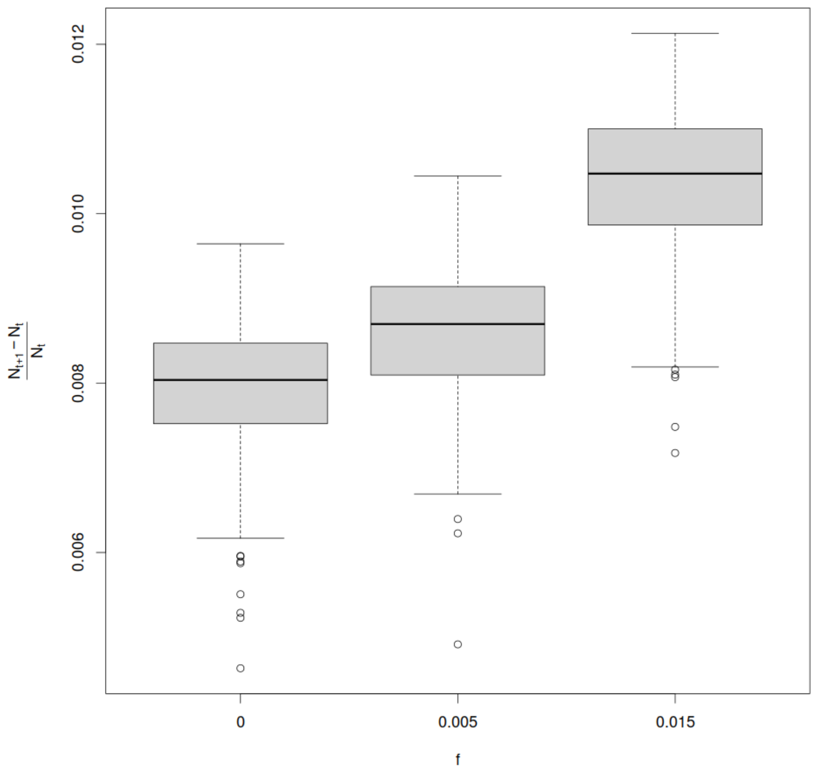

Fig. S2. Overall effective growth rate of the population measured from simulations explored in the main paper and grouped by growth benefit f_i. Growth rates are measured for both $p_{\text{location}} = 0$ and $p_{\text{location}} = 0.5$, total of 250 repetitions per box.

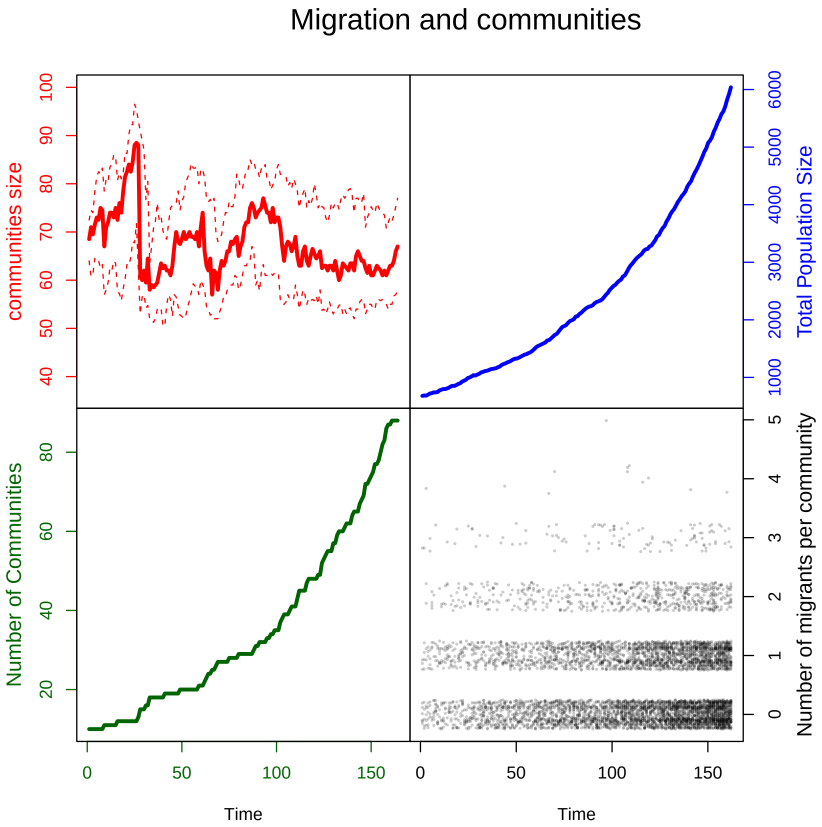

Fig. S3. Top-left: median and 75% interval for community sizes. Bottom-left: number of communities over time. Top-right: total population size. Bottom-right: number of migrants arriving at a new community at each time-step. Results are from a single simulation run with $\beta = -10$ and $f = 0.005$, which was terminated when the population reached 6,000 individuals to save computing time. All other parameters are consistent with those used in the main paper simulations. Although most communities typically receive one or no migrants, it is not uncommon to see instances with 3 or 4 migrants when the number of communities and population size increase. Fig. S6 illustrates the likelihood that these numbers of migrants will lead a community to adopt the migrants' trait variant.

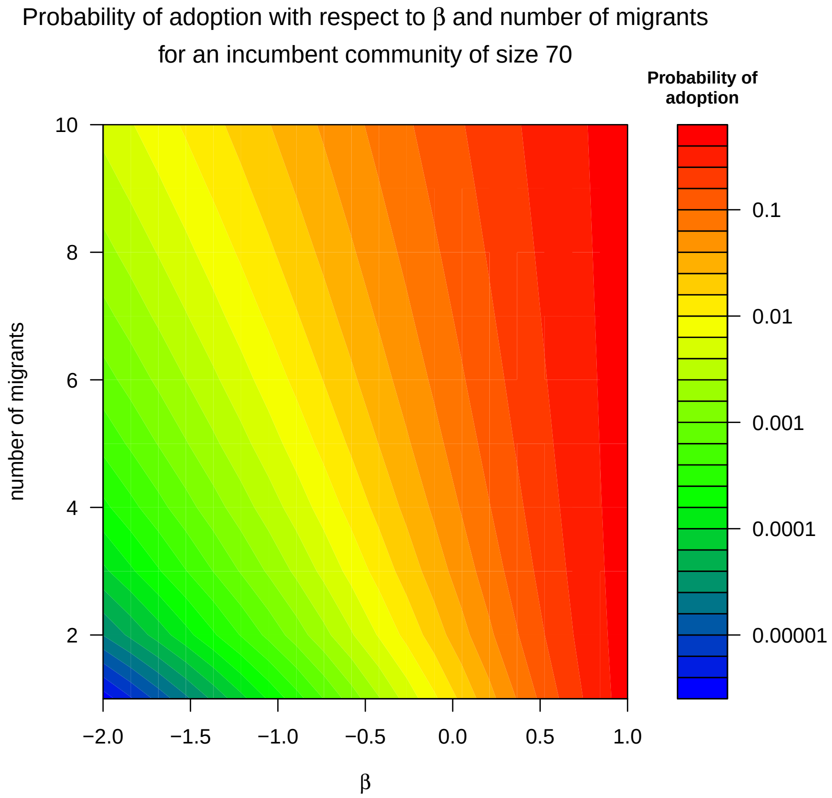

Fig. S4. Probability that a community of 70 individuals will adopt a trait from a migrant from another community for different settings of β. The probabilities are computed using Eq. (1) in the main text with $n = 70$ and $\beta \in [-2, 1]$. Colours represent powers of ten between 10^{-6} and 0.5. When $\beta = 1$, the number of migrants does not impact the probability of adoption greatly, and the community will randomly adopt the trait ($p_{a_i} = 0.5 \forall k_i$). When $\beta = 0$, the probability of adopting the trait variant from a single migrant is close to 0.014. This implies that even with only 10 communities, as at the beginning of all simulations, it is sufficient to wait, on average, 8 time steps to expect one of these communities to adopt the trait variant of a migrant. Although this may seem like a strong effect, it is important to consider that migrants may come from a community with the same technology, thus reducing the chance of adopting a new trait variant.

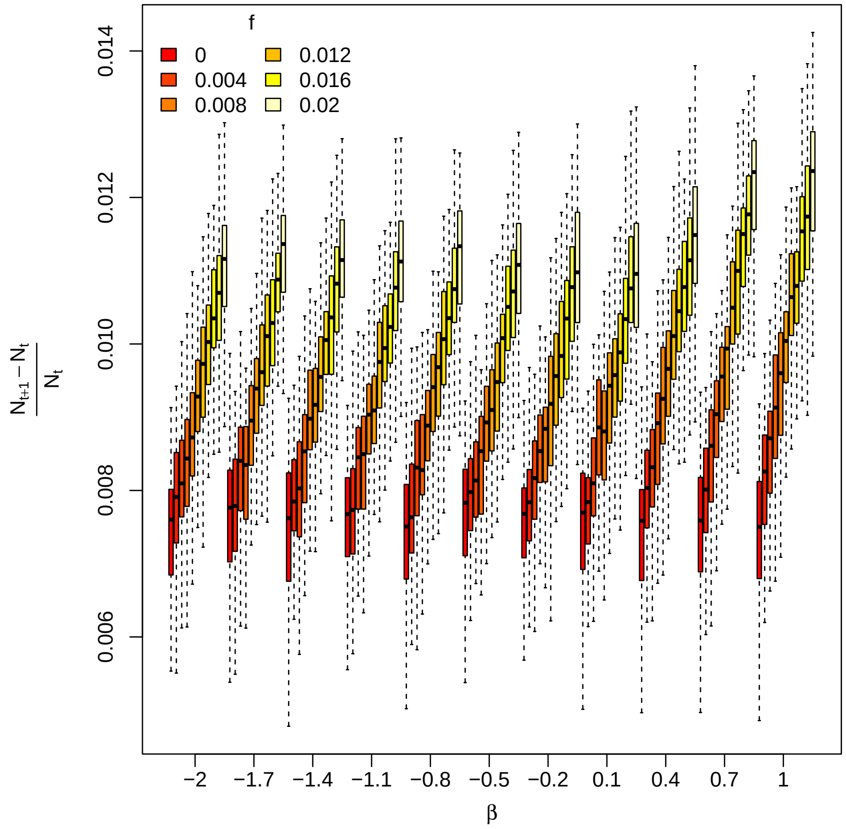

Fig. S5. Effective growth rate for 11 values of $f_i = f$ between 0 and 0.02 and 11 values of β between -2 and 1. Each box summarises the results of 100 simulations. Aside β and f, all other parameters are kept consistent with the one used in the main paper.

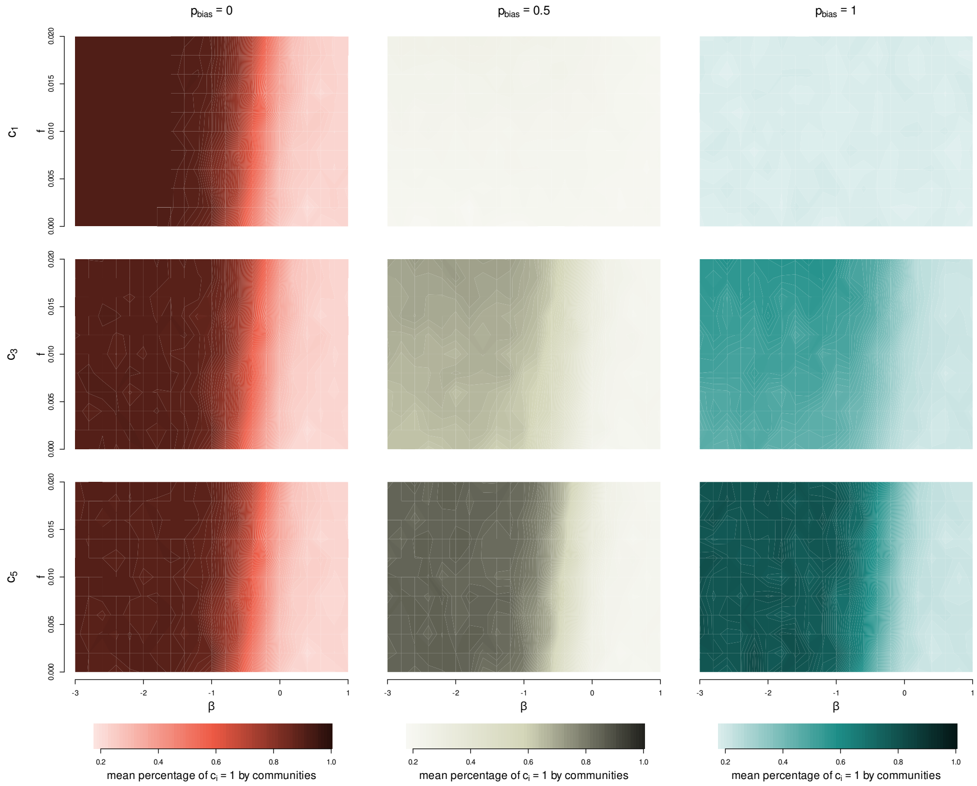

Fig. S6. Impact of $f_i = f$ and β on the mean proportion of individual carrying $c_i = 1$ in type B/C communities for $i = 1, 3, 5$ for the three different $p_{\text{bias}} = 0; 0.5; 1$. Colours represent the value of the mean proportion over 100 simulations for 11 value of f between 0 and 0.02 and 21 values of β between -3 and 1.

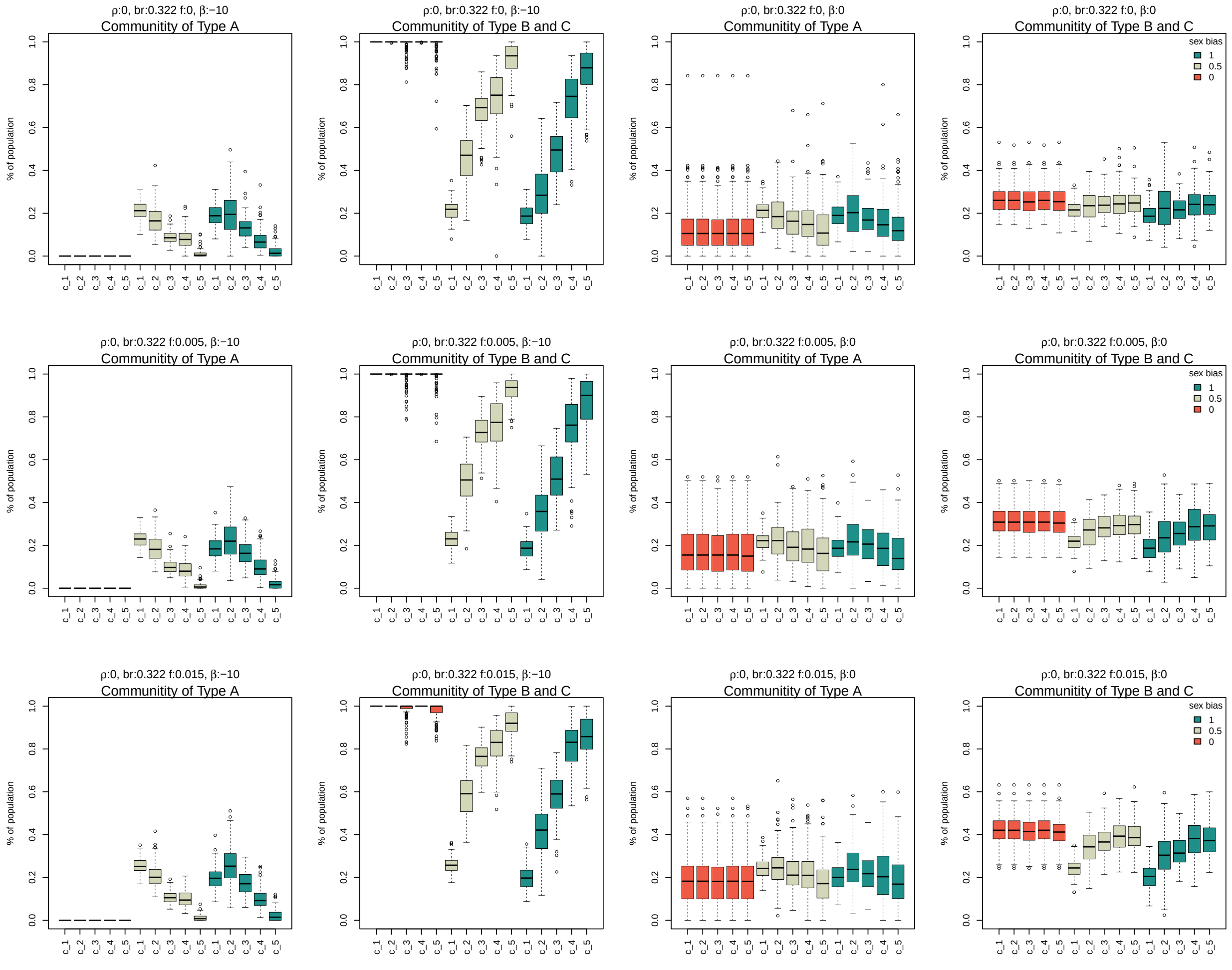

Fig. S7. Proportion of individuals in type A and B/C communities that possess $c_i = 1, i = 1, \ldots, 5$ at the end of each simulation for $f = f_1 = f_2 = f_3 = 0; 0.005; 0.015$, $p_{\text{location}} = 0$, $\beta = -10; 0$. The colour of the boxes represents different values of p_{bias} (red:0, grey:0.5, and blue:1).

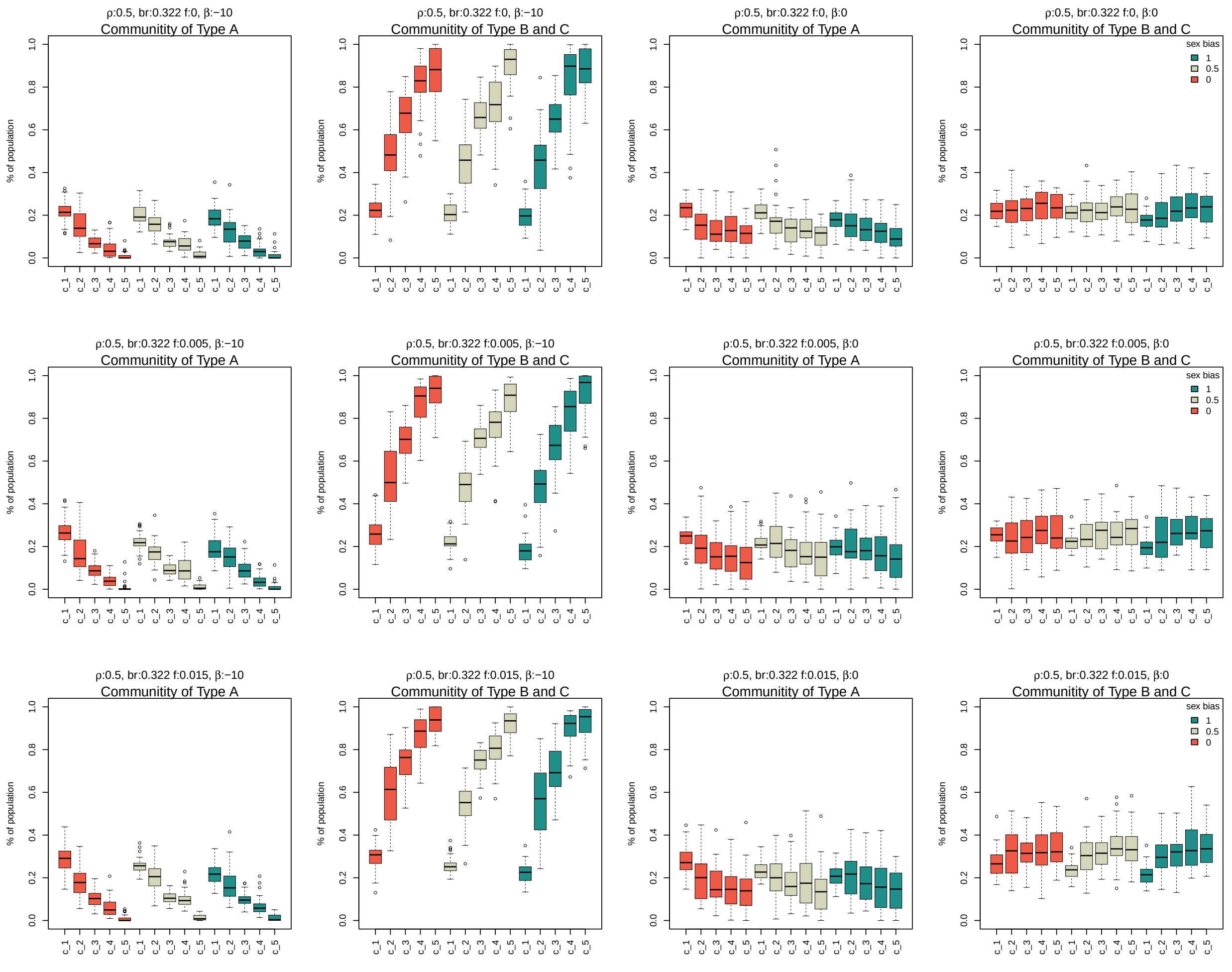

Fig. S8. Proportion of individuals in type A communities that possess $c_i = 1, i = 1, \ldots, 5$ (top row), proportion of individuals in type B and C communities that possess $c_i = 1, i = 1, \ldots, 5$ (bottom row) at the end of each simulation for $f = f_1 = f_2 = f_3 = 0.005; 0.015$, $\beta = -10; 0$, different transmission pathway labelled by $c_1, \ldots, c_5$ (see Table (1) in the main text), $p_{\text{bias}} = 0$ (colour red), $p_{\text{bias}} = 0.5$ (colour grey), $p_{\text{bias}} = 1$ (colour blue) and bilocality, i.e. $p_{\text{location}} = 0.5$.

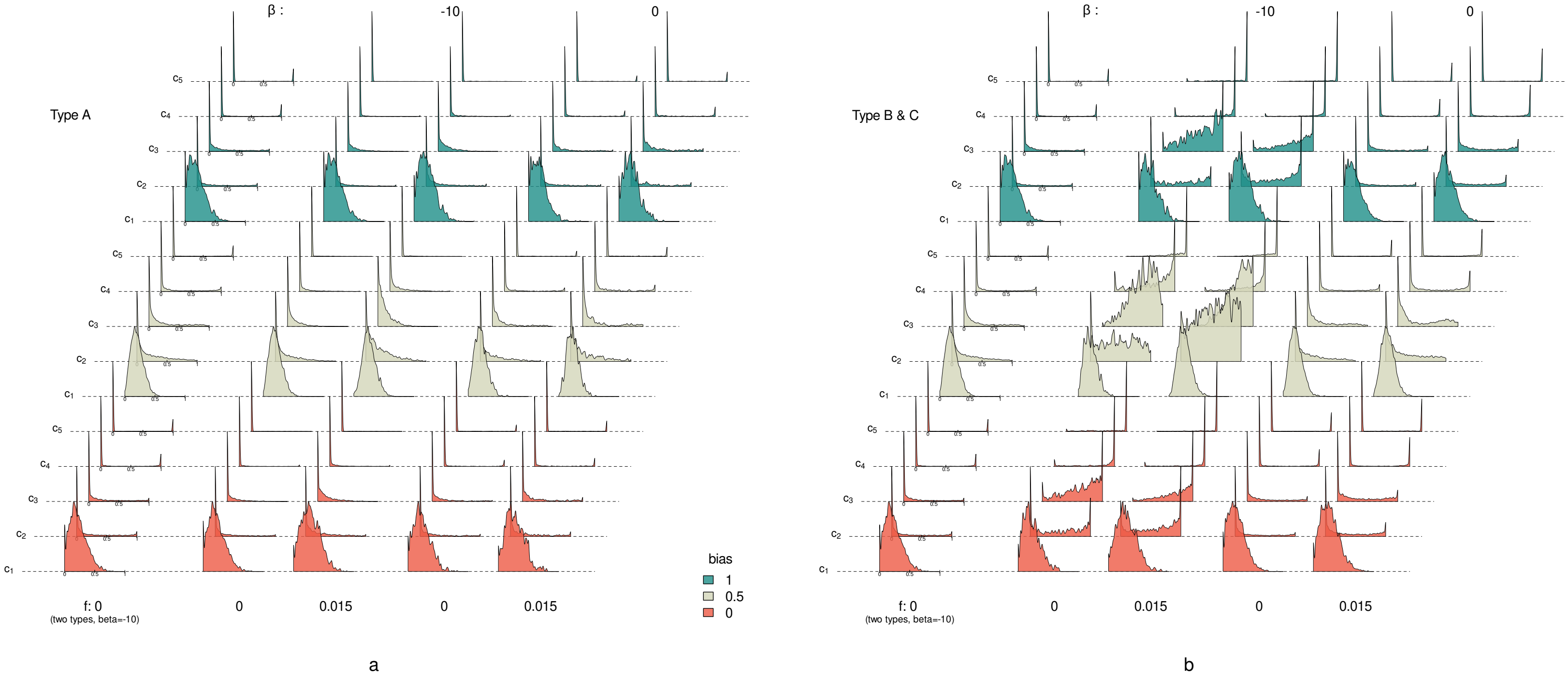

Fig. S9. Distributions of the proportion of individuals carrying $c_i = 1$ in type A (left panel) and type B/C (right panel) communities at the end of the simulation accumulated over 125 repetitions for $\beta = -10$ and $f_i = 0; 0.015$, $i = 1, 2, 3$ (second and third columns), $\beta = 0$ and $f_i = 0; 0.015$, $i = 1, 2, 3$ (fourth and fifth columns), labelled by different transmission pathways (see Table 1 in the main text), $p_{\text{bias}} = 0$ (colour red), $p_{\text{bias}} = 0.5$ (colour grey), $p_{\text{bias}} = 1$ (colour blue) and bilocality, i.e. $p_{\text{location}} = 0.5$.
